# Lamellar rotation surgery: a new procedure for repairing upper eyelid defects

**DOI:** 10.1186/s12886-018-0958-0

**Published:** 2018-11-07

**Authors:** Qingji Li

**Affiliations:** Department of Oculoplastic and Reconstructive Surgery, Tianjin Aier Eye Hospital, No.102, Fukang Road, Nankai District, Tianjin, 300190 People’s Republic of China

**Keywords:** Upper eyelid defect, Eyelid reconstruction, Lamellar rotation surgery

## Abstract

**Background:**

To report “Lamellar Rotation Surgery”,a new technique for repairing large and moderate full-thickness upper eyelid defects.

**Methods:**

A two-stage technique is described in which a vertical incision is made in the tarsus of the lower eyelid with elevation of the lateral posterior lamella while sparing the lower eyelid orbicularis and skin to be rotated superiorly to form the reconstructed posterior lamella of the upper eyelid. Additionally, a lateral periosteal flap is used to reconstruct the lateral canthal tendon, and a McGregor procedure is used to reconstruct the anterior lamella of the upper eyelid. The flap is divided during a second-stage surgery at 3 months. Three cases are described to showcase this technique.

**Results:**

Good functional and aesthetic results were achieved for the eyelids.

**Conclusions:**

This new procedure may help to address the challenge of repairing full-thickness defects of the upper eyelid.

**Trial registration:**

Registration number: ChiCTR1800018990, 20 Oct 2018, retrospectively registered.

**Electronic supplementary material:**

The online version of this article (10.1186/s12886-018-0958-0) contains supplementary material, which is available to authorized users.

## Background

Upper eyelid full-thickness defects are caused by tumour excision, trauma or congenital colobomas. Small defects (less than 33% of eyelid margin involvement) can be repaired by direct closure with superior cantholysis if necessary [1]. The most commonly used procedure for moderate defects (33–50% involvement) is the inverted semi-circular flap [1], and the Cutler-Beard flap [1–4] is used for large defects (over 50% involvement). The reconstruction of large upper eyelid full-thickness defects represents a challenge in ocular plastic surgery because of the complicated anatomy and function. Certain surgical procedures, including the Cutler-Beard flap and Mustarde lid switch [1, 5], are available, but all these procedures have limitations. We have developed a new method that could provide a useful solution to this challenging problem for both large and moderate defects. Three cases are described to showcase this technique.

## Methods

### Surgical technique

#### First-stage surgery (Figs. 1, 2 and 3)

We vertically incised the tarsus of the lower eyelid and disconnected the lateral inferior retractor, conjunctiva and lateral canthal tendon while sparing the lower eyelid orbicularis and skin. The lateral tarsus was rotated superiorly to reconstruct the posterior lamella of the upper eyelid. Additionally, two lateral periosteal flaps were used to reconstruct the lateral canthal tendon, and a McGregor procedure [6, 7] was used to reconstruct the anterior lamella of the upper eyelid.

**Fig. 1:**
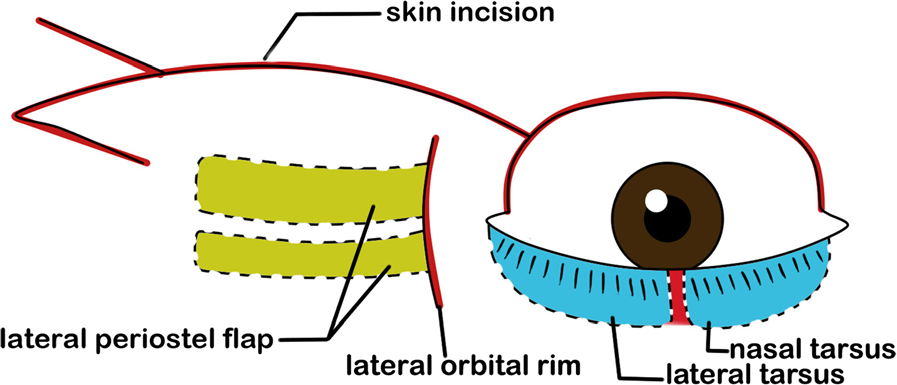
Diagrams to illustrate the designs of our procedure, illustrates procedures performed during the first-stage surgery

**Fig. 2:**
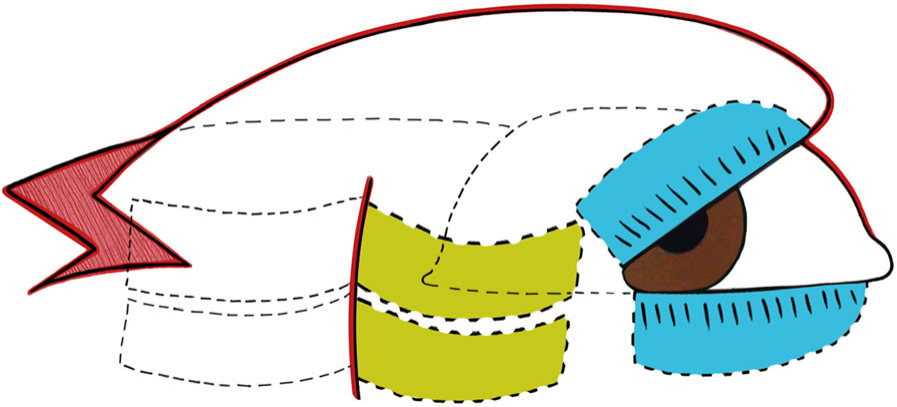
Diagrams to illustrate the designs of our procedure, illustrates rotation of the tarsus and skin flap and mobilisation of the lateral periosteal flap

**Fig. 3:**
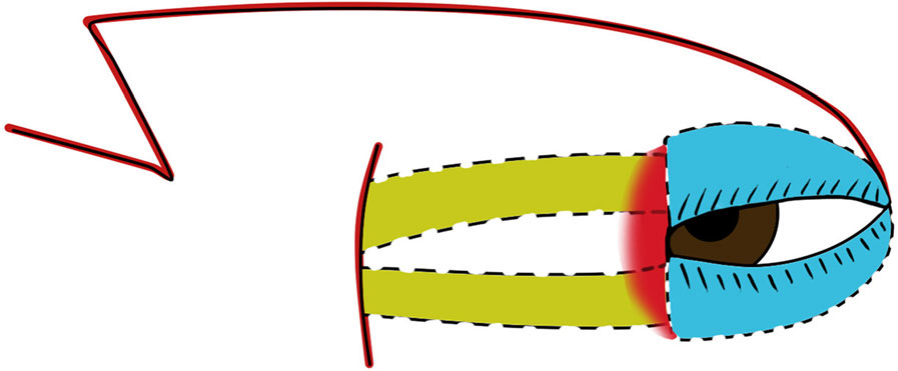
Diagrams to illustrate the designs of our procedure, illustrates the status when the surgery was finished

#### Second-stage surgery (Fig. 4)

The flap was divided during a second-stage surgery at 3 months.

**Fig. 4:**
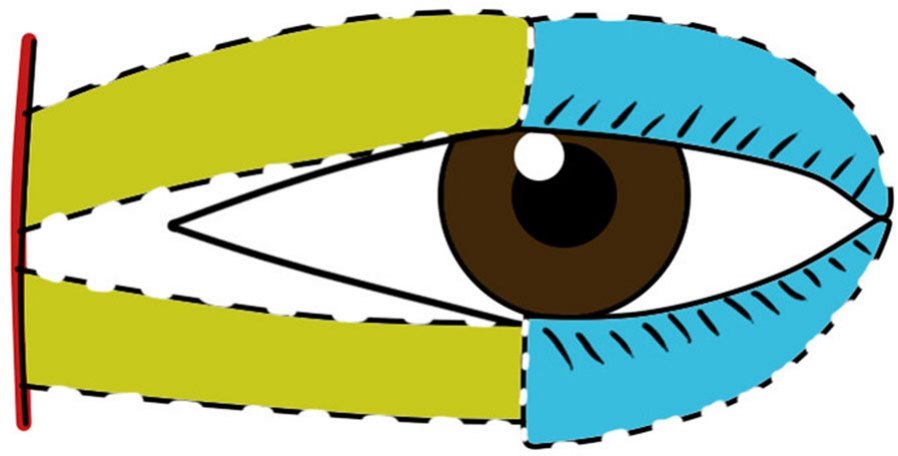
Diagrams to illustrate the designs of our procedure, illustrates the division of the flap 3 months later

### Case 1

**Fig. 5:**
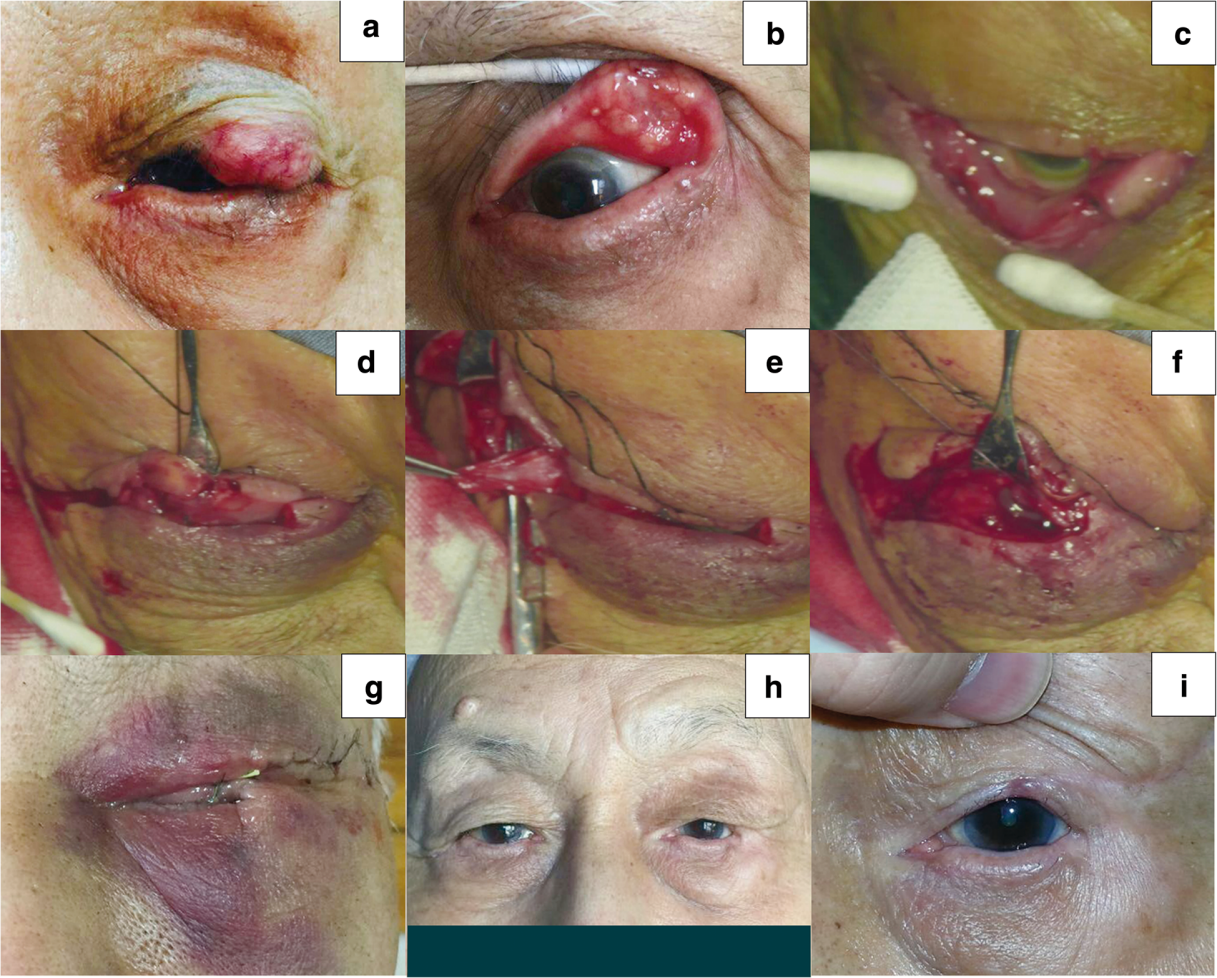
Case 1. **a** and **b** A large tumour on the left upper eyelid. **c** A large defect after excision. **d** The temporal tarsus of the lower eyelid with sparing of the orbicularis and eyelid skin after disconnection. **e** The lateral periosteal flap. **f** Connecting the rotated tarsus to the remnant nasal tarsus of the upper eyelid, levator, and lateral periosteal flap. **g** Status when the first stage of the surgery was complete. **h** Photograph at 18 months following division of the flap. **i** Details of **h**, also showing the rotated eyelashes that were retained

An 84-year-old man presented with a slow-growing mass on the left upper eyelid for one year with no treatment. On examination, a 30 × 25 mm hard mass involving the tarsus was observed (Fig. 5a and b). No other ophthalmologic abnormality was detected. We performed complete excision (Fig. 5c, approximately 75% defect) and eyelid reconstruction with our procedure. The histopathologic diagnosis was sebaceous gland carcinoma. We incised the skin along the mark according to the McGregor procedure and undermined the skin-orbicularis flap. Subsequently, we vertically incised the lower eyelid tarsus and disconnected the retractor and conjunctiva of the lateral tarsus, sparing the orbicularis and eyelid skin (Fig. 5d). We made two lateral periosteal flaps (Fig. 5e) and connected the inferior flap to the nasal tarsus of the lower eyelid. Then, we rotated the tarsus and connected it to the remnant nasal tarsus of the upper eyelid, levator and superior lateral periosteal flap (Fig. 5f). Finally, we sutured the skin-orbicularis flap (Fig. 5g). We divided the flap during a second stage 3 months later. Good functional and aesthetic results were achieved for the eyelid (Fig. 5h and i). The surgical video is available in Additional file 1.

### Case 2

**Fig. 6:**
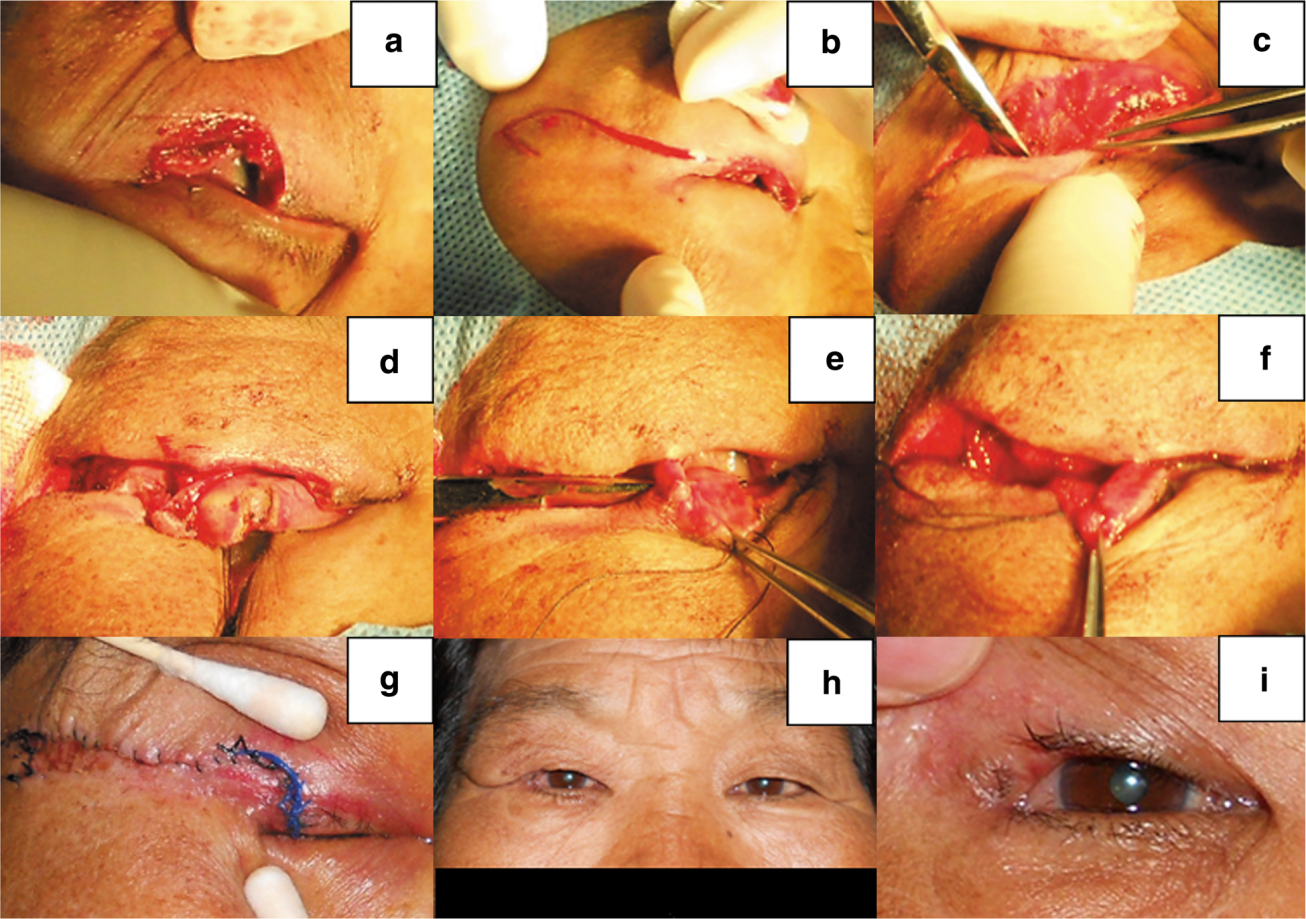
Case 2. **a** The defect after excision. **b** Incising the skin according to the McGregor procedure. **c** Undermining the skin-orbicularis flap. **d** Vertically incising the lower eyelid tarsus while sparing the anterior orbicularis muscle and skin. **e** Disconnecting the inferior retractor and conjunctiva. **f** Connecting the temporal tarsus of the lower eyelid with the remnant temporal tarsus of the upper eyelid. **g** Status when the first stage of the surgery was complete. **h** Photograph at 6 months following division of the flap. **i** Details of **h**

A 66-year-old woman presented with a recurrent mass on the right upper eyelid. She underwent local surgical excision twice at other clinics with no pathologic diagnosis. There was no evidence of regional lymph node involvement or distant metastases. On examination, a 10 × 7 mm hard mass involving the eyelid margin and tarsus was observed. After completely excising the mass (Fig. 6a, approximately 50% defect), we performed the procedures similar to Case 1 (Fig. 6b, c, d, e, f, g) except that we connected the temporal tarsus of the lower eyelid with the remnant temporal tarsus of the upper eyelid (Fig. 6f) and rotated the combined tarsus to reconstruct the posterior lamellar defect of the upper eyelid. The histopathologic diagnosis was sebaceous gland carcinoma. A satisfactory result was achieved (Fig. 6h and i).

### Case 3

**Fig. 7:**
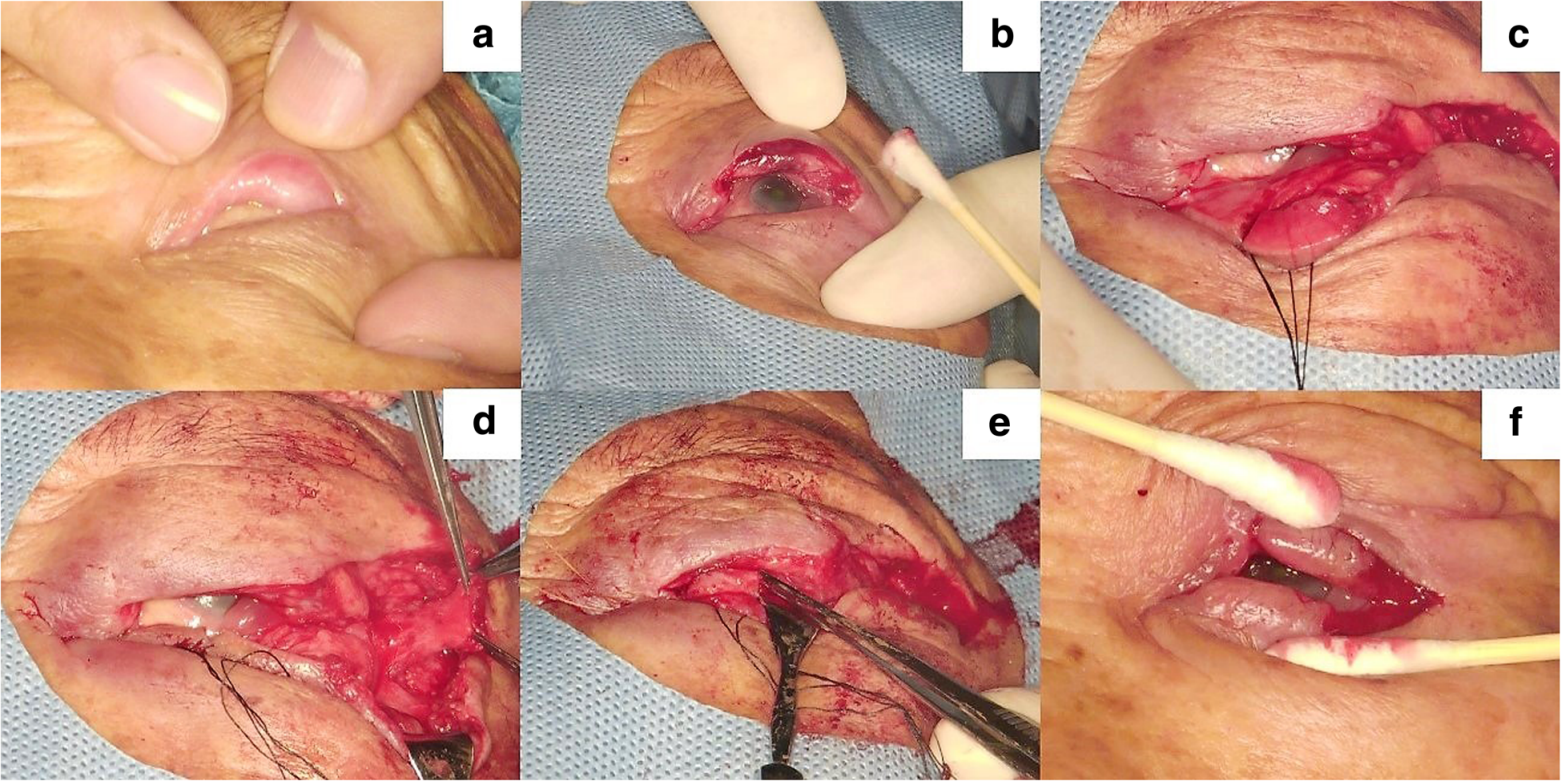
Case 3. **a** The left upper eyelid lacked the tarsus. **b** Excising the eyelid margin with histologic assurance of complete tumour removal. **c** The temporal tarsus of the lower eyelid sparing the orbicularis and eyelid skin. **d** The lateral periosteal flap. **e** The rotated tarsus. **f** Dividing the flap during the second stage of the surgery

A 78-year-old woman complained of a severe foreign body sensation after upper eyelid tumour (sebaceous gland carcinoma) excision at another clinic. On examination, the upper eyelid skin was found to overturn inwards and to be in contact with the cornea (Fig. 7a, eyelid margin defect approximately 90%). We performed similar procedures mainly to reconstruct the posterior lamella (Fig. 7b, c, d, e, f). The patient confirmed that the foreign body sensation had completely vanished after the surgery through telephone follow-up. However, she was unable to return to our clinic because of the long distance required for such travel. The video of the first stage surgery is available in Additional file 2.

## Discussion

The most commonly used technique to treat moderate upper eyelid defect is a lateral canthal tendon incision and semi-circular “Tenzel” flap [1, 8]. Large full-thickness upper eyelid defects are a challenge in ocular plastic surgery. Certain surgical procedures are available for reconstructing large full-thickness upper eyelid defects, but all these procedures have limitations. The Cutler-Beard procedure is likely the most popular in use, although the reconstructed upper eyelid is not sufficiently stable due to a lack of tarsus [1–4]. Moreover, the lanugo hairs present on the reconstructed upper eyelid may cause corneal irritation. The Mustarde lid switch procedure is not widely practised because of corneal irritation caused by the pedicle, among other reasons [1, 5]. Other techniques are mainly tarsoconjunctival substitutes, including hard palate [9], nasal septal chondromucosal [10], and free tarsomarginal grafts [11]. Hard palate and nasal septal chondromucosal grafts may be preferable as an option for lower eyelid rather than upper eyelid reconstruction because of corneal damage [1]. The survival risk and eyelash absence associated with free tarsomarginal grafts are mentioned in some studies [11].

The aim and novelty of our technique was the use of the lower eyelid tarsus, periosteal flap, and temporal skin-orbicularis flap to reconstruct an upper eyelid defect. We considered the lower eyelid and periorbital skin as an integral whole. We rotated them integrally to reconstruct the defect but treated each lamella differently and delicately. Consequently, we named this new procedure *lamellar rotation surgery*. We vertically incised the lower eyelid tarsus and disconnected the inferior retractor and conjunctiva, sparing the anterior orbicularis muscle and skin. As a result, the temporal part of the tarsus attained a high degree of flexibility and adequate nutrition from the intact orbicularis muscle and eyelid skin. The lateral periosteal flap was used not only to form a new lateral canthal tendon but also to reconstruct the posterior lamella to support the eyelid tissue.

The reconstructed upper eyelid should be more stable after lamellar rotation surgery than after the Cutler-Beard procedure because of the rotated tarsus. The pedicle of the Mustarde lid switch procedure may irritate the cornea because the intact tarsus of the lower eyelid maintains a degree of rigidity [1, 5], whereas our procedure could avoid this limitation due to the disconnected, flexible tarsus. In addition, the junction of the rotated flap and lower eyelid will be elongated because it comprises orbicularis skin. The flexibility of the upper eyelid will also improve after the first-stage surgery.

For a total upper eyelid defect, our procedure can provide a new eyelid margin to prevent upper eyelid skin contact with the cornea. The smooth surface and glands of the tarsus can also benefit the cornea.

A decrease in the length of the horizontal palpebral fissure and two-stage surgery are the limitations of our procedure. This procedure may also result in amblyopia if applied to children.

Our study also has the following limitations: a small sample size, non-comparative design because of the rare incidence, and short follow-up because of long-distance travel and economy.

Although we have treated only three cases, we believe that our procedure can help to address the challenge of upper eyelid defects and should complement existing surgical methods. However, all three cases were elderly individuals with tumours, and we have no experience with trauma or congenital colobomas. Further studies with larger sample sizes are required to further validate the findings of this study and to modify the procedure, for example, if there is a need for the superior lateral periosteal flap.

## Conclusions

The new procedure may help to address the challenge of repairing full-thickness defects of the upper eyelid.

## Additional files

Additional file 1: The surgical video of Case 1. (MOV 16113 kb)

Additional file 2: The video of the first stage surgery of Case 3. (MOV 17381 kb)
